# Development and Validation of the Chronic Disease Population Risk Tool (CDPoRT) to Predict Incidence of Adult Chronic Disease

**DOI:** 10.1001/jamanetworkopen.2020.4669

**Published:** 2020-06-04

**Authors:** Ryan Ng, Rinku Sutradhar, Kathy Kornas, Walter P. Wodchis, Joykrishna Sarkar, Randall Fransoo, Laura C. Rosella

**Affiliations:** 1 Dalla Lana School of Public Health, Division of Epidemiology, University of Toronto, Toronto, Ontario, Canada; 2 ICES, Toronto, Ontario, Canada; 3 Institute of Health Policy, Management and Evaluation, University of Toronto, Toronto, Ontario, Canada; 4 Trillium Health Partners’ Institute for Better Health, Mississauga, Ontario, Canada; 5 Manitoba Centre for Health Policy, University of Manitoba, Winnipeg, Manitoba, Canada

## Abstract

**Importance:**

Predicting chronic disease incidence for the population provides a comprehensive picture to health policy makers of their jurisdictions’ overall future chronic disease burden. However, no population-based risk algorithm exists for estimating the risk of first major chronic disease.

**Objective:**

To develop and validate the Chronic Disease Population Risk Tool (CDPoRT), a population risk algorithm that predicts the 10-year incidence of the first major chronic disease in the adult population.

**Design, Setting, and Participants:**

In this cohort study, CDPoRT was developed and validated with 6 cycles of the Canadian Community Health Survey, linked to administrative data from January 2000 to December 2014. Development and internal validation (bootstrap and split sample) of CDPoRT occurred in Ontario, Canada, from June 2018 to April 2019 followed by external validation in Manitoba from May 2019 to July 2019. The study cohorts included 133 991 adults (≥20 years) representative of the Ontario and Manitoba populations who did not have a history of major chronic disease.

**Exposures:**

Predictors were routinely collected risk factors from the Canadian Community Health Survey, such as sociodemographic factors (eg, age), modifiable lifestyle risk factors (ie, alcohol consumption, cigarette smoking, unhealthy diet, and physical inactivity), and other health-related factors (eg, body mass index).

**Main Outcomes and Measures:**

Six major chronic diseases were considered, as follows: congestive heart failure, chronic obstructive pulmonary disease, diabetes, myocardial infarction, lung cancer, and stroke. Sex-specific CDPoRT algorithms were developed with a Weibull model. Model performance was evaluated with measures of overall predictive performance (eg, Brier score), discrimination (eg, Harrell C index), and calibration (eg, calibration curves).

**Results:**

The Ontario cohort (n = 118 747) was younger (mean [SD] age, 45.6 [16.1] vs 46.3 [16.4] years), had more immigrants (23 808 [20.0%] vs 1417 [10.7%]), and had a lower mean (SD) body mass index (26.9 [5.1] vs 27.7 [5.4]) than the Manitoba cohort (n = 13 244). During development, the full and parsimonious CDPoRT models had similar Brier scores (women, 0.087; men, 0.091), Harrell C index values (women, 0.779; men, 0.783), and calibration curves. A simple version consisting of cigarette smoking, age, and body mass index performed slightly worse than the other versions (eg, Brier score for women, 0.088; for men, 0.092). Internal validation showed consistent performance across models, and CDPoRT performed well during external validation. For example, the female parsimonious version had C index values for bootstrap, split sample, and external validation of 0.778, 0.776, and 0.752, respectively.

**Conclusions and Relevance:**

In this study, CDPoRT provided accurate, population-based risk estimates for the first major chronic disease.

## Key Points

**Question:**

Can a prognostic model using lifestyle risk factors accurately predict the incidence of the first major chronic disease (ie, congestive heart failure, chronic obstructive pulmonary disease, diabetes, myocardial infarction, lung cancer, or stroke) at a population level?

**Findings:**

In this cohort study, sex-specific prognostic models were developed and validated, demonstrating high overall predictive performance, discrimination, and calibration during development, internal validation, and external validation.

**Meaning:**

Using routinely collected risk factor information, the Chronic Disease Population Risk Tool exhibited reproducibility and geographic transportability for predicting the 10-year incidence of the first major chronic disease at the population level.

## Teaser

This study reports the development and validation of a population risk algorithm that predicts the 10-year incidence of the first major chronic disease in an adult population.

## Introduction

The high prevalence of chronic disease is a worldwide health issue, with 6.7 billion people worldwide living with a noncommunicable disease resulting in substantial years of life lost.^1,2^ Chronic disease prevalence has increased over time,^2^ which can be attributed to an aging population and improvements in chronic disease management.^2,3,4^ As a result of this increase and an increase in multimorbidity (the presence of multiple chronic conditions),^5^ high costs are borne by the health care system. The total direct and indirect costs of chronic conditions are estimated to be 19.6% of the total US gross domestic product ($3.7 trillion).^6^ A tool that predicts the future burden of multiple chronic diseases on the health care system would be invaluable to support health policy makers in their planning and prevention efforts.

Primary prevention is an ideal strategy to address the chronic disease burden.^7^ Well-established evidence shows that 4 modifiable lifestyle risk factors, ie, alcohol consumption, cigarette smoking, unhealthy diet, and physical inactivity, are associated with more than two-thirds of the incidence of cancer, cardiovascular disease, chronic respiratory disease, and diabetes combined.^7^ Despite the evidence, preventing or delaying chronic disease onset with primary prevention strategies is difficult.^7^ One problem is that no straightforward way exists to predict the incidence of chronic disease in the population, which has been stated as a need from health policy makers.^8^ One solution for population prediction is with a population risk algorithm.^8,9^ These algorithms predict the risk of an outcome (eg, cardiovascular disease) like clinical risk algorithms do (eg, Framingham Risk Score),^10^ but they are designed to make predictions representative of the overall population (instead of individual-level prediction) by using routinely collected risk factor information (eg, smoking) from a population-level data source (eg, the National Household Interview Survey).^9^

There have been related attempts to predict future chronic disease burden. An existing clinical risk score that predicts the incidence of multiple chronic diseases was developed in a specific private health care insurance patient population,^11^ so its generalizability to the general population is unknown. There are also population risk algorithms that predict the incidence of individual chronic diseases (eg, diabetes).^9,12,13,14,15^ However, to our knowledge, no risk algorithm exists that can predict the incidence of the first major chronic disease at the population level.

To meet this need, this study’s objective was to create the Chronic Disease Population Risk Tool (CDPoRT). The tool uses routinely collected, self-reported information on lifestyle risk factors from population health surveys to predict the first incidence of 1 of 6 major chronic diseases (ie, congestive heart failure, chronic obstructive pulmonary disease, diabetes, lung cancer, myocardial infarction, and stroke including transient ischemic attack) during a 10-year period in the general adult population. Furthermore, CDPoRT was validated with a variety of techniques to quantify its reproducibility and transportability.

## Methods

This section summarizes the methods, with additional details available in a published analytic protocol.^16^ Overall, the protocol was adhered to with some modifications. First, the maximum follow-up was reduced from 15 to 10 years to eliminate model instability caused by very few individuals with 15 years of follow-up. Second, an additional exclusion omitted respondents with missing predictor values (exceptions were body mass index [calculated as weight in kilograms divided by height in meters squared] and income). Third, the Weibull model replaced the Royston-Parmar model because the log cumulative baseline hazard function approximated a straight line, indicating a hazard function with a Weibull distribution (eFigure 1 in the Supplement). Fourth, simple and parsimonious versions of CDPoRT were developed and validated along with the proposed model (ie, full version). The versions show the contributions of the predictors toward model performance. Fifth, a sensitivity analysis was added to understand how CDPoRT performed within the competing risks framework. Sixth, a sensitivity analysis in which each chronic disease was modeled individually and then combined to understand the contributions of each disease to overall chronic disease risk was not completed because of computational nonconvergence.

The study was approved by the research ethics boards of the University of Toronto, Sunnybrook Health Sciences Centre, and University of Manitoba. Informed patient consent was waived because ICES and Manitoba Centre for Health Policy are prescribed entities under the Personal Health Information Protection Act and Personal Health Information Act, respectively. This report was written in accordance with the Transparent Reporting of a Multivariable Prediction Model for Individual Prognosis or Diagnosis (TRIPOD) reporting guideline for prediction model development and validation.

### Data Sources

The Canadian Community Health Survey represents 98% of the Canadian population aged 12 years and older^17^ and contains information on sociodemographic characteristics, health status, and health determinants. The survey was linked to administrative data from 2 Canadian provinces. For development and internal validation in Ontario, the data were linked with unique encoded identifiers and analyzed at ICES. For external validation in Manitoba, data held at the Manitoba Centre for Health Policy were used.

### Participants

All Canadian Community Health Survey respondents in Ontario from the first 6 cycles (1.1 [2000], 2.1 [2003], 3.1 [2005], 4.1 [2007], 2009/2010, and 2011/2012) who consented to administrative data linkage were eligible. Respondents were excluded if they were younger than 20 years at interview; had a history of congestive heart failure, chronic obstructive pulmonary disease, diabetes, lung cancer, myocardial infarction, or stroke according to self-report or diagnosis from administrative data; or had missing predictor information (except body mass index and income). The earliest response was used for individuals with multiple survey responses. The Manitoba validation cohort consisted of respondents from Canadian Community Health Survey cycles 3.1, 4.1, 2009/2010, and 2011/2012 who met the same exclusion criteria.

### Chronic Disease Outcomes

The chronic diseases were congestive heart failure, chronic obstructive pulmonary disease, diabetes, lung cancer, myocardial infarction, and stroke including transient ischemic attack. They were chosen according to their prevalence,^5^ established links with lifestyle risk factors,^7^ associations with morbidity and mortality,^1,2,18,19^ and importance to the intended users of CDPoRT.^8^ All diseases were identified from the administrative data with validated algorithms,^20,21,22,23,24,25^ with vital statistics supplementing additional outcomes based on cause of death.

### Predictors

Sixteen candidate predictors were identified from the Canadian Community Health Survey in accordance with well-established associations,^18,26,27,28^ subject matter expertise, user input, the group’s experiences with population risk algorithms,^9,12,14,29,30,31,32^ and variable availability across cycles. The self-reported predictors were collected once as of the interview. The predictors were sociodemographic predictors (eg, age, ethnicity, immigration status, household income, education, marital status), modifiable lifestyle risk factors (eg, alcohol consumption, cigarette smoking, daily fruit and vegetable consumption, physical activity), and other health-related factors (eg, asthma, body mass index, high blood pressure, household secondhand smoke, self-rated health, life stress). Ethnicity was categorized as white or visible minority according to Canadian Community Health Survey response categories. Ethnicity was included as a predictor because the incidence of risk of chronic diseases varies by ethnicity.^33,34,35^

### Study Design

**Figure 1.**
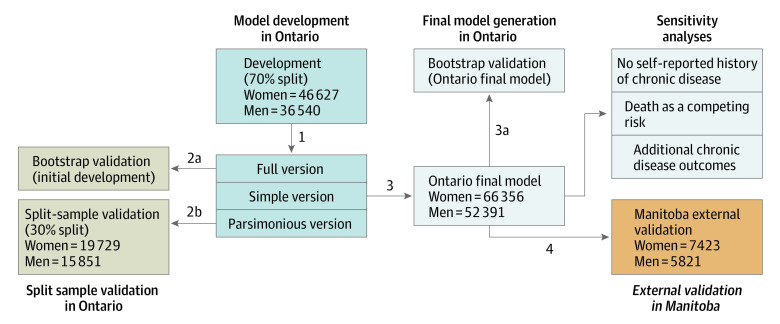
Steps Used in the Development and Validation of the Chronic Disease Population Risk Tool

Sex-specific CDPoRT prognostic models were created because the biological underpinnings of chronic disease vary by sex.^36,37,38,39,40^ The prognostic models were developed and internally validated in Ontario and externally validated in Manitoba (Figure 1). For model development in Ontario, CDPoRT was developed in a 70% random sample of the respondents (step 1). Full, simple, and parsimonious versions of CDPoRT were developed (details in the “Model Specification” section). For the split sample validation in Ontario, internal validation via the bootstrap (1000 samples with replacement) was conducted with the 70% split (step 2a). The remaining 30% was used for split sample validation (step 2b). For final model generation in Ontario, in accordance with inefficiencies of the split sample approach,^41,42,43^ the entire Ontario cohort was combined to estimate the final Ontario CDPoRT models (step 3). Internal validation via bootstrapping was also conducted (step 3). For external validation in Manitoba, the full, simple, and parsimonious versions of the final Ontario CDPoRT models were validated externally within Manitoba (step 4).

### Model Specification

A subset of the 16 candidate predictors was selected for the full version based on predictive performance. Predictive performance was evaluated with measures of overall predictive performance (eg, Nagelkerke *R*^2^, Brier score), discrimination (eg, Harrell C index, time-specific discrimination slopes), and calibration (time-specific calibration curves, calibration intercepts, and calibration slopes); definitions of each measure are shown in eTable 1 in the Supplement. As an additional check of predictor importance, an analysis of variance with partial χ^2^ test with prespecified statistical significance level of *P* < .05 was also used to confirm predictor importance. All time-specific statistics were calculated at 10 years. Candidate predictors that did not improve predictive performance were removed. Prespecified forms of the predictors were used (eTable 2 in the Supplement), and after sequential selection, alternative specifications were examined. For both sexes, cigarette smoking was respecified. Age was also respecified as a restricted cubic spline for both sexes. Education was respecified in the female model, whereas income was respecified in the male model. The final predictor specifications of the full version (eTable 2 in the Supplement) were used in the simple and parsimonious versions. Two-way interactions and cross-classification were also examined between age, smoking, and body mass index.

The simple version included only influential predictors. These were predictors that considerably reduced discrimination (ie, Harrell C index <0.7 [poor discrimination]^44^) or calibration (ie, major deviation of the calibration plot) when excluded from the full model. The parsimonious version struck a balance between the simple and full versions because it had more predictors than the simple version and fewer than the full version, but had predictive performance similar to that of the full version. The parsimonious version was constructed with forward selection, starting from the simple version in which the remaining predictors were added individually until predictive performance was similar to that of the full version.

### Statistical Analysis

Between January 2000 and December 2014, respondents were observed from the Canadian Community Health Survey interview until chronic disease incidence with censoring on death, 10 years of follow-up, or study end. The models were estimated with a Weibull model. Proportional hazards were assessed with scaled Schoenfeld residuals. By incorporating Canadian Community Health Survey weights, all estimates were representative of the population. Variances were calculated from bootstrap survey weights via the percentile bootstrap.^45,46^

A sensitivity analysis was performed among individuals who did not self-report a previous chronic disease but were excluded because of diagnosis from the administrative data. This analysis shows how CDPoRT performs when administrative data cannot be accessed to exclude individuals with chronic disease. A second sensitivity analysis modeled death as a competing risk to determine how the subdistribution hazard ratios compared with the cause-specific ones. A third sensitivity analysis examined CDPoRT’s performance when 9 additional chronic diseases were considered as outcomes (ie, cardiac arrhythmia, chronic coronary syndrome, Crohn disease or colitis, dementia, osteoporosis, arthritis, rheumatoid arthritis, osteoarthritis, and renal failure).^5^

Data were cleaned with SAS Enterprise Guide version 7.1 (SAS Institute), whereas R version 3.1.2 (R Project for Statistical Computing) was used for model development and internal validation. SAS was used for external validation. Statistical significance was set at *P* < .05, and tests were 1-tailed.

## Results

The baseline characteristics of the 70% Ontario development cohort (n = 83 167), the 30% Ontario split sample validation cohort (n = 35 580), and the Manitoba external validation cohort (n = 13 244) are provided in Table 1. Cohort exclusions appear in eFigure 2 in the Supplement. Major case-mix differences were that the Manitoba cohort was older (mean [SD] age, 46.3 [16.4] years vs 45.6 [16.1] years), had fewer immigrants (1417 [10.7%] vs 23 808 [20.0%]), and had higher mean (SD) body mass index (27.7 [5.4] vs 26.9 [5.1]) compared with the combined Ontario cohort. The 10-year risk of first chronic disease was greater in Manitoba (women, 12.5%; men, 13.1%) vs Ontario (women, 11.2%; men, 11.8%).

**Table 1.** Baseline Characteristics of the Development and Validation Cohorts Stratified by Sex

Variable	No. (%)					
	Development (70% split)	Split sample validation (30% split)	All Ontario		Manitoba external validation	
**Women**						
No.	46 627	19 729	66 356		7423	
Follow-up time, mean (SD), y	6.93 (2.88)	6.90 (2.82)	6.92 (2.85)		7.40 (2.76)	
Age, y						
Mean (SD)	46.59 (16.74)	46.55 (16.82)	46.58 (16.76)		47.19 (17.72)	
20-34	13 338 (28.6)	5711 (28.9)	19 049 (28.7)		2262 (30.5)	
35-44	9898 (21.2)	4197 (21.3)	14 095 (21.2)		1300 (17.5)	
45-54	8127 (17.4)	3383 (17.1)	11 510 (17.3)		1273 (17.1)	
55-64	7566 (16.2)	3155 (16.0)	10 721 (16.2)		1239 (16.7)	
65-74	4675 (10.0)	1935 (9.8)	6610 (10.0)		732 (9.9)	
75-84	2431 (5.2)	1098 (5.6)	3529 (5.3)		482 (6.5)	
≥85	592 (1.3)	250 (1.3)	842 (1.3)		135 (1.8)	
Alcohol consumption						
None	29 299 (62.8)	12 429 (63.0)	41 728 (62.9)		5066 (68.2)	
Light	8280 (17.8)	3524 (17.9)	11 804 (17.8)		1090 (14.7)	
Moderate	7736 (16.6)	3209 (16.3)	10 945 (16.5)		1051 (14.2)	
Heavy	1312 (2.8)	567 (2.9)	1879 (2.8)		216 (2.9)	
Cigarette smoking						
Never	19 032 (40.8)	7978 (40.4)	27 010 (40.7)		2942 (39.6)	
Always occasional	629 (1.3)	284 (1.4)	913 (1.4)		103 (1.4)	
Former daily, now occasional	1300 (2.8)	545 (2.8)	1845 (2.8)		216 (2.9)	
Daily	7926 (17.0)	3369 (17.1)	11 295 (17.0)		1327 (17.9)	
Former occasional	7439 (16.0)	3207 (16.3)	10 646 (16.0)		1122 (15.1)	
Former daily	10 301 (22.1)	4346 (22.0)	14 647 (22.1)		1713 (23.1)	
Fruit and vegetable consumption, times/d						
<3	11 152 (23.9)	4623 (23.4)	15 775 (23.8)		1605 (21.6)	
3-6	21 806 (46.8)	9276 (47.0)	31 082 (46.8)		2584 (34.8)	
>6	13 669 (29.3)	5830 (29.6)	19 499 (29.4)		1150 (15.5)	
Physical activity, quartile						
1, lowest	10 928 (23.4)	4600 (23.3)	15 528 (23.4)		1568 (21.1)	
2	12 785 (27.4)	5394 (27.3)	18 179 (27.4)		1986 (26.8)	
3	11 554 (24.8)	4901 (24.8)	16 455 (24.8)		1885 (25.4)	
4, highest	11 360 (24.4)	4834 (24.5)	16 194 (24.4)		1984 (26.7)	
Visible minority	5606 (12.0)	2375 (12.0)	7981 (12.0)		1119 (15.1)	
Immigration status						
Canadian born	37 158 (79.7)	15 822 (80.2)	52 980 (79.8)		6642 (89.5)	
Recent immigrant	1901 (4.1)	763 (3.9)	2664 (4.0)		230 (3.1)	
Nonrecent immigrant	7568 (16.2)	3144 (15.9)	10 712 (16.1)		551 (7.4)	
Household income, quintile						
1, lowest	5525 (11.8)	2334 (11.8)	7859 (11.8)		1433 (19.3)	
2	6391 (13.7)	2620 (13.3)	9011 (13.6)		1444 (19.5)	
3	8868 (19.0)	3795 (19.2)	12 663 (19.1)		1443 (19.4)	
4	11 082 (23.8)	4893 (24.8)	15 975 (24.1)		1288 (17.4)	
5, highest	11 417 (24.5)	4674 (23.7)	16 091 (24.2)		1208 (16.3)	
Unknown income	3344 (7.2)	1413 (7.2)	4757 (7.2)		607 (8.2)	
Education						
<Secondary school	6121 (13.1)	2657 (13.5)	8778 (13.2)		1271 (17.1)	
Secondary school	9080 (19.5)	3944 (20.0)	13 024 (19.6)		1435 (19.3)	
Any postsecondary education	31 426 (67.4)	13 128 (66.5)	44 554 (67.1)		4717 (63.5)	
Marital status						
Single, never married	8887 (19.1)	3780 (19.2)	12 667 (19.1)		1447 (19.5)	
Domestic partner, ie, married or common law	28 032 (60.1)	11 807 (59.8)	39 839 (60.0)		4540 (61.2)	
Widowed, separated, or divorced	9708 (20.8)	4142 (21.0)	13 850 (20.9)		1436 (19.3)	
Asthma	3925 (8.4)	1664 (8.4)	5589 (8.4)		561 (7.6)	
BMI						
Mean (SD)	26.45 (5.47)	26.45 (5.50)	26.45 (5.48)		27.16 (5.77)	
<18.5	656 (1.4)	299 (1.5)	955 (1.4)		97 (1.3)	
18.5 to <25.0	19 446 (41.7)	8210 (41.6)	27 656 (41.7)		2765 (37.2)	
25.0 to <30.0	13 883 (29.8)	5876 (29.8)	19 759 (29.8)		2384 (32.1)	
30.0 to <35.0	6041 (13.0)	2576 (13.1)	8617 (13.0)		1084 (14.6)	
35.0 to <40.0	2044 (4.4)	863 (4.4)	2907 (4.4)		401 (5.4)	
≥40.0	1015 (2.2)	438 (2.2)	1453 (2.2)		232 (3.1)	
Unknown	3542 (7.6)	1467 (7.4)	5009 (7.5)		460 (6.2)	
High blood pressure	6992 (15.0)	2976 (15.1)	9968 (15.0)		1239 (16.7)	
Household secondhand smoke	4048 (8.7)	1703 (8.6)	5751 (8.7)		764 (10.3)	
Self-rated health						
Poor or fair	3858 (8.3)	1659 (8.4)	5517 (8.3)		661 (8.9)	
Good	11 805 (25.3)	4967 (25.2)	16 772 (25.3)		2032 (27.4)	
Very good or excellent	30 964 (66.4)	13 103 (66.4)	44 067 (66.4)		4730 (63.7)	
Life stress						
Not at all stressful	4432 (9.5)	1859 (9.4)	6291 (9.5)		709 (9.6)	
Not very stressful	11 144 (23.9)	4625 (23.4)	15 769 (23.8)		1921 (25.9)	
A bit stressful	20 137 (43.2)	8534 (43.3)	28 671 (43.2)		3265 (44)	
Quite a bit or extremely stressful	10 914 (23.4)	4711 (23.9)	15 625 (23.5)		1528 (20.6)	
**Men**						
No.	36 540	15 851		52 391		5821
Follow-up time, mean (SD), y	6.93 (2.82)	6.87 (2.78)		6.91 (2.80)		7.35 (2.79)
Age, y						
Mean (SD)	44.35 (15.14)	44.43 (15.17)		44.38 (15.15)		45.11 (15.60)
20-34	10 922 (29.9)	4628 (29.2)		15 550 (29.7)		1769 (30.4)
35-44	9053 (24.8)	4027 (25.4)		13 080 (25.0)		1210 (20.8)
45-54	6934 (19.0)	3055 (19.3)		9989 (19.1)		1134 (19.5)
55-64	5531 (15.1)	2347 (14.8)		7878 (15.0)		984 (16.9)
65-74	2927 (8.0)	1261 (8.0)		4188 (8.0)		488 (8.4)
75-84	1006 (2.8)	456 (2.9)		1462 (2.8)		204 (3.5)
≥85	167 (0.5)	77 (0.5)		244 (0.5)		32 (0.5)
Alcohol consumption						
None	14 714 (40.3)	6408 (40.4)		21 122 (40.3)		2786 (47.9)
Light	5851 (16.0)	2466 (15.6)		8317 (15.9)		806 (13.8)
Moderate	11 216 (30.7)	4816 (30.4)		16 032 (30.6)		1544 (26.5)
Heavy	4759 (13.0)	2161 (13.6)		6920 (13.2)		685 (11.8)
Cigarette smoking						
Never	10 450 (28.6)	4504 (28.4)		14 954 (28.5)		1762 (30.3)
Always occasional	761 (2.1)	313 (2.0)		1074 (2.0)		125 (2.1)
Former daily, now occasional	1199 (3.3)	534 (3.4)		1733 (3.3)		192 (3.3)
Daily	8107 (22.2)	3598 (22.7)		11 705 (22.3)		1246 (21.4)
Former occasional	6435 (17.6)	2814 (17.8)		9249 (17.7)		925 (15.9)
Former daily	9588 (26.2)	4088 (25.8)		13 676 (26.1)		1571 (27.0)
Fruit and vegetable consumption, times/d						
<3	14 163 (38.8)	6304 (39.8)		20 467 (39.1)		2042 (35.1)
3-6	16 714 (45.7)	7049 (44.5)		23 763 (45.4)		1691 (29.1)
>6	5663 (15.5)	2498 (15.8)		8161 (15.6)		458 (7.9)
Physical activity, quartile						
1, lowest	8795 (24.1)	3789 (23.9)		12 584 (24.0)		1238 (21.3)
2	9698 (26.5)	4200 (26.5)		13 898 (26.5)		1513 (26.0)
3	9019 (24.7)	4026 (25.4)		13 045 (24.9)		1331 (22.9)
4, highest	9028 (24.7)	3836 (24.2)		12 864 (24.6)		1739 (29.9)
Visible minority	4608 (12.6)	2041 (12.9)		6649 (12.7)		835 (14.3)
Immigration status						
Canadian born	29 256 (80.1)	12 703 (80.1)		41 959 (80.1)		5185 (89.1)
Recent immigrant	1461 (4.0)	613 (3.9)		2074 (4.0)		188 (3.2)
Nonrecent immigrant	5823 (15.9)	2535 (16.0)		8358 (16.0)		448 (7.7)
Household income, quintile						
1, lowest	3009 (8.2)	1335 (8.4)		4344 (8.3)		726 (12.5)
2	3836 (10.5)	1673 (10.6)		5509 (10.5)		970 (16.7)
3	6167 (16.9)	2681 (16.9)		8848 (16.9)		1171 (20.1)
4	9837 (26.9)	4242 (26.8)		14 079 (26.9)		1310 (22.5)
5, highest	11 874 (32.5)	5166 (32.6)		17 040 (32.5)		1349 (23.2)
Unknown income	1817 (5.0)	754 (4.8)		2571 (4.9)		295 (5.1)
Education						
<Secondary school	4811 (13.2)	2130 (13.4)		6941 (13.2)		1146 (19.7)
Secondary school	6896 (18.9)	3078 (19.4)		9974 (19.0)		1156 (19.9)
Any postsecondary education	24 833 (68.0)	10 643 (67.1)		35 476 (67.7)		3519 (60.5)
Marital status						
Single, never married	9501 (26.0)	4132 (26.1)		13 633 (26.0)		1565 (26.9)
Domestic partner, ie, married or common law	22 795 (62.4)	9865 (62.2)		32 660 (62.3)		3590 (61.7)
Widowed, separated, or divorced	4244 (11.6)	1854 (11.7)		6098 (11.6)		666 (11.4)
Asthma	1971 (5.4)	928 (5.9)		2899 (5.5)		343 (5.9)
BMI						
Mean (SD)	27.44 (4.59)	27.39 (4.62)		27.43 (4.60)		27.99 (5.08)
<18.5	238 (0.7)	105 (0.7)		343 (0.7)		34 (0.6)
18.5 to <25.0	11 004 (30.1)	4833 (30.5)		15 837 (30.2)		1568 (26.9)
25.0 to <30.0	16 090 (44.0)	6989 (44.1)		23 079 (44.1)		2588 (44.5)
30.0 to <35.0	6386 (17.5)	2723 (17.2)		9109 (17.4)		1165 (20.0)
35.0 to <40.0	1480 (4.1)	615 (3.9)		2095 (4.0)		303 (5.2)
≥40.0	522 (1.4)	231 (1.5)		753 (1.4)		125 (2.1)
Unknown	820 (2.2)	355 (2.2)		1175 (2.2)		38 (0.7)
High blood pressure	4798 (13.1)	2151 (13.6)		6949 (13.3)		846 (14.5)
Household secondhand smoke	3527 (9.7)	1552 (9.8)		5079 (9.7)		625 (10.7)
Self-rated health						
Poor or fair	2688 (7.4)	1124 (7.1)		3812 (7.3)		427 (7.3)
Good	9547 (26.1)	4194 (26.5)		13 741 (26.2)		1698 (29.2)
Very good or excellent	24 305 (66.5)	10 533 (66.5)		34 838 (66.5)		3696 (63.5)
Life stress						
Not at all stressful	4198 (11.5)	1951 (12.3)		6149 (11.7)		637 (10.9)
Not very stressful	8890 (24.3)	3758 (23.7)		12 648 (24.1)		1610 (27.7)
A bit stressful	15 660 (42.9)	6699 (42.3)		22 359 (42.7)		2501 (43.0)
Quite a bit or extremely stressful	7792 (21.3)	3443 (21.7)		11 235 (21.4)		1073 (18.4)

Abbreviation: BMI, body mass index (calculated as weight in kilograms divided by height in meters squared).

### CDPoRT Development

The full versions of the CDPoRT for women and men consisted of 11 predictors, with their coefficients and hazard ratios listed in Table 2. No interactions or cross-classifications improved the predictive performance of the full versions. Age, smoking, and body mass index had the greatest influence on predictive performance, and thereby were retained in the simple version for both sexes (Table 2); for example, they had high predictor importance based on large χ^2^ values of 1117 for age, 631 for smoking, and 453 for body mass index for women and 1166 for age, 337 for smoking, and 314 for body mass index for men. For both sexes, the parsimonious version contained the same 9 predictors, ie, alcohol consumption, cigarette smoking, fruit and vegetable consumption, age, visible minority, asthma, body mass index, high blood pressure, and self-rated health (Table 2). There were no gross violations of the proportionality assumption.

**Table 2.** Chronic Disease Population Risk Tool for Women and Men Proportional Hazards Coefficients and Hazard Ratios for the Full, Simple, and Parsimonious Versions, by Ontario Cohort

Variable	Cohort			
	Development		Entire Ontario	
	ln[HR] (95% CI)	HR	ln[HR] (95% CI)	HR
**Full version for women**				
Weibull parameters				
Scale, σ	0.8924	NA	0.8869	NA
Shape, γ	1.1206	NA	1.1275	NA
Intercept	−4.311 (−4.71 to −3.91)	0.0134	−4.345 (−4.68 to −4.00)	0.0130
Alcohol consumption				
Light	NA	NA	NA	NA
Heavy	0.41 (0.12 to 0.70)	1.51	0.24 (−0.01 to 0.49)	1.27
Moderate	0.09 (−0.07 to 0.24)	1.09	0.10 (−0.03 to 0.23)	1.11
None	0.34 (0.22 to 0.47)	1.41	0.32 (0.22 to 0.41)	1.37
Cigarette smoking				
Never	NA	NA	NA	NA
Always occasional	0.36 (−0.09 to 0.81)	1.44	0.27 (−0.11 to 0.65)	1.31
Former daily, now occasional	0.61 (0.34 to 0.89)	1.84	0.61 (0.38 to 0.84)	1.84
Daily	1.05 (0.93 to 1.18)	2.87	1.05 (0.95 to 1.15)	2.86
Former occasional	−0.16 (−0.30 to −0.02)	0.85	−0.16 (−0.27 to −0.04)	0.86
Former daily	0.20 (0.09 to 0.32)	1.23	0.20 (0.10 to 0.30)	1.22
Fruit and vegetable consumption, times/d				
<3	NA	NA	NA	NA
3-6	−0.06 (−0.17 to 0.05)	0.94	−0.06 (−0.15 to 0.03)	0.94
>6	−0.12 (−0.25 to 0.00)	0.88	−0.12 (−0.22 to −0.01)	0.89
Age, spline term				
1	0.13 (0.11 to 0.16)	1.14	0.13 (0.11 to 0.15)	1.14
2	−0.26 (−0.34 to −0.18)	0.77	−0.25 (−0.32 to −0.18)	0.78
3	0.51 (0.34 to 0.69)	1.67	0.51 (0.36 to 0.66)	1.66
Visible minority	0.37 (0.23 to 0.51)	1.44	0.34 (0.22 to 0.46)	1.41
Postsecondary education	−0.06 (−0.16 to 0.03)	0.94	−0.10 (−0.19 to −0.02)	0.90
Marital status				
Domestic partner	NA	NA	NA	NA
Single, never married	0.05 (−0.11 to 0.21)	1.06	0.07 (−0.06 to 0.21)	1.08
Widowed, separated, or divorced	0.09 (−0.02 to 0.20)	1.09	0.08 (−0.01 to 0.18)	1.09
Asthma	0.36 (0.22 to 0.50)	1.43	0.37 (0.25 to 0.50)	1.45
BMI				
<18.5	NA	NA	NA	NA
18.5 to <25.0	−0.33 (−0.76 to 0.10)	0.72	−0.18 (−0.56 to 0.19)	0.83
25.0 to <30.0	0.32 (0.20 to 0.45)	1.38	0.37 (0.27 to 0.47)	1.45
30.0 to <35.0	0.61 (0.47 to 0.75)	1.84	0.61 (0.50 to 0.73)	1.85
35.0 to <40.0	1.01 (0.80 to 1.22)	2.74	1.03 (0.86 to 1.20)	2.81
≥40.0	1.24 (1.01 to 1.47)	3.46	1.16 (0.97 to 1.36)	3.20
Unknown	0.47 (0.32 to 0.62)	1.59	0.43 (0.31 to 0.56)	1.54
High blood pressure	0.35 (0.24 to 0.46)	1.42	0.33 (0.23 to 0.42)	1.39
Self-rated health				
Good	NA	NA	NA	NA
Poor or fair	0.23 (0.09 to 0.37)	1.26	0.19 (0.08 to 0.31)	1.21
Very good or excellent	−0.14 (−0.24 to −0.03)	0.87	−0.19 (−0.27 to −0.10)	0.83
Life stress				
Not at all stressful	NA	NA	NA	NA
A bit stressful	−0.02 (−0.17 to 0.12)	0.98	0.00 (−0.13 to 0.13)	1.00
Not very stressful	−0.13 (−0.29 to 0.02)	0.88	−0.10 (−0.23 to 0.02)	0.90
Quite a bit or extremely stressful	−0.03 (−0.20 to 0.13)	0.97	0.02 (−0.13 to 0.16)	1.02
**Simple version for women**				
Weibull parameters	NA	NA	NA	NA
Scale, σ	0.9000	NA	0.8940	NA
Shape, γ	1.1111	NA	1.1186	NA
Intercept	−4.083 (−4.42 to −3.75)	0.0169	−4.210 (−4.49 to −3.93)	0.0148
Cigarette smoking				
Never	NA	NA	NA	NA
Always occasional	0.29 (−0.17 to 0.74)	1.33	0.18 (−0.20 to 0.56)	1.20
Former daily, now occasional	0.51 (0.23 to 0.78)	1.66	0.50 (0.27 to 0.73)	1.65
Daily	0.99 (0.87 to 1.12)	0.73	1.00 (0.90 to 1.10)	0.74
Former occasional	−0.31 (−0.45 to −0.17)	2.70	−0.30 (−0.42 to −0.18)	2.72
Former daily	0.04 (−0.07 to 0.16)	1.04	0.05 (−0.04 to 0.14)	1.05
Age, spline term				
1	0.13 (0.11 to 0.15)	1.14	0.12 (0.10 to 0.14)	1.13
2	−0.25 (−0.33 to −0.17)	0.78	−0.24 (−0.30 to −0.17)	0.79
3	0.52 (0.35 to 0.69)	1.68	0.49 (0.35 to 0.64)	1.64
BMI				
<18.5	NA	NA	NA	NA
18.5 to <25.0	−0.21 (−0.63 to 0.21)	2.12	−0.07 (−0.44 to 0.30)	2.14
25.0 to <30.0	0.39 (0.26 to 0.51)	3.42	0.43 (0.33 to 0.52)	3.55
30.0 to <35.0	0.75 (0.62 to 0.89)	4.32	0.76 (0.65 to 0.87)	4.08
35.0 to <40.0	1.23 (1.02 to 1.43)	1.47	1.27 (1.10 to 1.43)	1.53
≥40.0	1.46 (1.25 to 1.68)	0.81	1.41 (1.23 to 1.59)	0.93
Unknown	0.54 (0.39 to 0.69)	1.71	0.52 (0.39 to 0.64)	1.67
**Parsimonious version for women**				
Weibull parameters				
Scale, σ	0.8922	NA	0.8864	NA
Shape, γ	1.1208	NA	1.1282	NA
Intercept	−4.384 (−4.75 to −4.01)	0.0125	−4.424 (−4.73 to −4.11)	0.0120
Alcohol consumption				
Light	NA	NA	NA	NA
Heavy	0.42 (0.13 to 0.71)	1.52	0.26 (0.01 to 0.51)	1.29
Moderate	0.08 (−0.07 to 0.24)	1.09	0.10 (−0.03 to 0.23)	1.10
None	0.35 (0.23 to 0.47)	1.42	0.33 (0.23 to 0.43)	1.39
Cigarette smoking				
Never	NA	NA	NA	NA
Always occasional	0.38 (−0.07 to 0.82)	1.46	0.29 (−0.09 to 0.66)	1.33
Former daily, now occasional	0.62 (0.34 to 0.89)	1.85	0.61 (0.38 to 0.84)	1.84
Daily	1.07 (0.95 to 1.2)	2.93	1.08 (0.97 to 1.18)	2.93
Former occasional	−0.16 (−0.3 to −0.02)	0.85	−0.16 (−0.28 to −0.04)	0.85
Former daily	0.20 (0.08 to 0.32)	1.23	0.20 (0.10 to 0.30)	1.22
Fruit and vegetable consumption, times/d				
<3	NA	NA	NA	NA
3-6	−0.07 (−0.17 to 0.04)	0.94	−0.07 (−0.17 to 0.02)	0.93
>6	−0.14 (−0.26 to −0.01)	0.87	−0.14 (−0.24 to −0.03)	0.87
Age, spline term				
1	0.13 (0.11 to 0.16)	1.14	0.13 (0.11 to 0.15)	1.14
2	−0.26 (−0.34 to −0.18)	0.77	−0.25 (−0.31 to −0.18)	0.78
3	0.52 (0.35 to 0.69)	1.68	0.51 (0.36 to 0.66)	1.66
Visible minority	0.37 (0.23 to 0.51)	1.44	0.34 (0.22 to 0.46)	1.41
Asthma	0.36 (0.22 to 0.5)	1.43	0.38 (0.26 to 0.50)	1.46
BMI				
<18.5	NA	NA	NA	NA
18.5 to <25.0	−0.32 (−0.74 to 0.11)	0.73	−0.18 (−0.55 to 0.19)	0.83
25.0 to <30.0	0.33 (0.2 to 0.45)	1.39	0.37 (0.27 to 0.47)	1.45
30.0 to <35.0	0.61 (0.47 to 0.75)	1.85	0.62 (0.50 to 0.73)	1.86
35.0 to <40.0	1.02 (0.8 to 1.23)	2.76	1.04 (0.87 to 1.21)	2.83
≥40.0	1.24 (1.02 to 1.47)	3.47	1.17 (0.98 to 1.36)	3.22
Unknown	0.47 (0.32 to 0.62)	1.60	0.44 (0.31 to 0.57)	1.55
High blood pressure	0.35 (0.24 to 0.46)	1.42	0.33 (0.24 to 0.42)	1.39
Self-rated health				
Good	NA	NA	NA	NA
Poor or fair	0.24 (0.11 to 0.38)	1.27	0.21 (0.10 to 0.33)	1.24
Very good or excellent	−0.14 (−0.25 to −0.04)	0.87	−0.19 (−0.28 to −0.10)	0.82
**Full version for men**				
Weibull parameters				
Scale, σ	0.8422	NA	0.8496	NA
Shape, γ	1.1874	NA	1.1770	NA
Intercept	−2.405 (−3.35 to −1.46)	0.0903	−3.183 (−3.92 to −2.45)	0.0415
Alcohol consumption				
Light	NA	NA	NA	NA
Heavy	−0.21 (−0.39 to −0.03)	0.81	−0.12 (−0.26 to 0.02)	0.89
Moderate	−0.05 (−0.19 to 0.09)	0.95	−0.01 (−0.13 to 0.11)	0.99
None	0.19 (0.06 to 0.33)	1.21	0.18 (0.06 to 0.30)	1.20
Cigarette smoking				
Never	NA	NA	NA	NA
Always occasional	0.14 (−0.35 to 0.63)	1.15	0.05 (−0.33 to 0.43)	1.05
Former daily, now occasional	0.33 (0.06 to 0.61)	1.40	0.35 (0.12 to 0.58)	1.42
Daily	0.86 (0.73 to 1.00)	2.37	0.83 (0.72 to 0.94)	2.30
Former occasional	0.04 (−0.13 to 0.22)	1.04	0.01 (−0.13 to 0.16)	1.01
Former daily	0.20 (0.08 to 0.33)	1.23	0.16 (0.05 to 0.27)	1.18
Fruit and vegetable consumption, times/d				
<3	NA	NA	NA	NA
3-6	−0.13 (−0.23 to −0.02)	0.88	−0.08 (−0.16 to 0.01)	0.93
>6	−0.22 (−0.39 to −0.06)	0.80	−0.16 (−0.30 to −0.03)	0.85
Age, spline term				
1	0.24 (0.18 to 0.29)	1.27	0.19 (0.15 to 0.23)	1.21
2	−0.63 (−0.90 to −0.37)	0.53	−0.42 (−0.63 to −0.21)	0.66
3	1.57 (0.74 to 2.40)	4.81	0.93 (0.26 to 1.60)	2.54
4	−1.04 (−1.84 to −0.24)	0.35	−0.48 (−1.15 to 0.18)	0.62
Visible minority	0.31 (0.16 to 0.45)	1.36	0.27 (0.15 to 0.39)	1.31
Household income				
Not low	NA	NA	NA	NA
Low	0.12 (−0.06 to 0.29)	1.12	0.11 (−0.04 to 0.27)	1.12
Unknown	0.08 (−0.13 to 0.30)	1.09	0.13 (−0.04 to 0.31)	1.14
Asthma	0.33 (0.14 to 0.51)	1.39	0.27 (0.11 to 0.44)	1.32
BMI				
<18.5	NA	NA	NA	NA
18.5 to <25.0	0.53 (−0.04 to 1.10)	1.69	0.42 (−0.07 to 0.92)	1.53
25.0 to <30.0	0.04 (−0.09 to 0.18)	1.04	0.14 (0.03 to 0.25)	1.15
30.0 to <35.0	0.51 (0.36 to 0.66)	1.66	0.57 (0.44 to 0.69)	1.77
35.0 to <40.0	0.87 (0.67 to 1.07)	2.38	0.98 (0.81 to 1.16)	2.67
≥40.0	1.16 (0.85 to 1.46)	3.18	1.19 (0.94 to 1.44)	3.28
Unknown	0.36 (0.14 to 0.59)	1.44	0.36 (0.17 to 0.55)	1.43
High blood pressure	0.39 (0.27 to 0.52)	1.48	0.36 (0.26 to 0.46)	1.43
Self-rated health				
Good	NA	NA	NA	NA
Poor or fair	0.12 (−0.03 to 0.27)	1.12	0.11 (−0.01 to 0.24)	1.12
Very good or excellent	−0.33 (−0.43 to −0.22)	0.72	−0.29 (−0.38 to −0.20)	0.75
Life stress				
Not at all stressful	NA	NA	NA	NA
A bit stressful	−0.01 (−0.17 to 0.14)	0.99	−0.03 (−0.16 to 0.10)	0.97
Not very stressful	−0.13 (−0.29 to 0.03)	0.88	−0.08 (−0.21 to 0.05)	0.92
Quite a bit or extremely stressful	−0.11 (−0.28 to 0.06)	0.89	−0.13 (−0.27 to 0.01)	0.88
**Simple version for men**				
Weibull parameters				
Scale, σ	0.8522	NA	0.8571	NA
Shape, γ	1.1735	NA	1.1667	NA
Intercept	−2.629 (−3.52 to −1.74)	0.0721	−3.343 (−4.03 to −2.65)	0.0353
Cigarette smoking				
Never	NA	NA	NA	NA
Always occasional	0.10 (−0.41 to 0.61)	1.11	0.03 (−0.37 to 0.42)	1.03
Former daily, now occasional	0.27 (0.01 to 0.54)	1.31	0.34 (0.12 to 0.56)	1.40
Daily	0.87 (0.74 to 1.00)	2.38	0.84 (0.73 to 0.95)	2.33
Former occasional	−0.03 (−0.19 to 0.14)	0.97	−0.04 (−0.18 to 0.10)	0.96
Former daily	0.14 (0.01 to 0.26)	1.15	0.12 (0.01 to 0.22)	1.12
Age, spline term				
1	0.23 (0.18 to 0.29)	1.26	0.18 (0.14 to 0.23)	1.20
2	−0.59 (−0.86 to −0.32)	0.56	−0.39 (−0.60 to −0.17)	0.68
3	1.42 (0.59 to 2.25)	4.13	0.83 (0.16 to 1.51)	2.30
4	−0.89 (−1.70 to −0.08)	0.41	−0.39 (−1.06 to 0.28)	0.68
BMI				
<18.5	NA	NA	NA	NA
18.5 to <25.0	0.66 (0.09 to 1.23)	1.94	0.53 (0.04 to 1.02)	1.70
25.0 to <30.0	0.55 (0.40 to 0.70)	1.03	0.12 (0.48 to 0.72)	1.13
30.0 to <35.0	1.00 (0.81 to 1.20)	1.73	0.60 (0.93 to 1.27)	1.82
35.0 to <40.0	1.42 (1.14 to 1.70)	2.73	1.10 (1.19 to 1.65)	3.01
≥40.0	0.03 (−0.11 to 0.16)	4.14	1.42 (0.01 to 0.23)	4.13
Unknown	0.34 (0.10 to 0.57)	1.40	0.32 (0.13 to 0.51)	1.38
**Parsimonious version for men**				
Weibull parameters				
Scale, σ	0.8433	NA	0.8504	NA
Shape, γ	1.1858	NA	1.1759	NA
Intercept	−2.485 (−3.39 to −1.58)	0.0833	−3.279 (−3.99 to −2.57)	0.0377
Alcohol consumption				
Light	NA	NA	NA	NA
Heavy	−0.21 (−0.39 to −0.03)	0.81	−0.12 (−0.26 to 0.03)	0.89
Moderate	−0.05 (−0.19 to 0.09)	0.95	−0.01 (−0.13 to 0.11)	0.99
None	0.20 (0.07 to 0.34)	1.22	0.19 (0.07 to 0.30)	1.21
Cigarette smoking				
Never	NA	NA	NA	NA
Always occasional	0.15 (−0.34 to 0.63)	1.16	0.06 (−0.32 to 0.45)	1.06
Former daily, now occasional	0.34 (0.07 to 0.61)	1.40	0.36 (0.13 to 0.59)	1.43
Daily	0.87 (0.73 to 1.00)	2.38	0.84 (0.73 to 0.95)	2.31
Former occasional	0.04 (−0.13 to 0.21)	1.04	0.01 (−0.13 to 0.15)	1.01
Former daily	0.20 (0.08 to 0.33)	1.23	0.16 (0.06 to 0.27)	1.18
Fruit and vegetable consumption, times/d				
<3	NA	NA	NA	NA
3-6	−0.13 (−0.24 to −0.02)	0.88	−0.08 (−0.16 to 0.01)	0.92
>6	−0.23 (−0.39 to −0.06)	0.80	−0.17 (−0.30 to −0.03)	0.85
Age, spline term				
1	0.23 (0.18 to 0.29)	1.26	0.19 (0.15 to 0.23)	1.21
2	−0.62 (−0.89 to −0.36)	0.54	−0.41 (−0.62 to −0.20)	0.67
3	1.54 (0.71 to 2.37)	4.66	0.91 (0.24 to 1.58)	2.47
4	−1.01 (−1.81 to −0.21)	0.36	−0.46 (−1.13 to 0.20)	0.63
Visible minority	0.32 (0.18 to 0.46)	1.38	0.29 (0.17 to 0.41)	1.33
Asthma	0.32 (0.14 to 0.51)	1.38	0.27 (0.11 to 0.43)	1.31
BMI				
<18.5	NA	NA	NA	NA
18.5 to <25.0	0.53 (−0.04 to 1.10)	1.70	0.43 (−0.07 to 0.93)	1.54
25.0 to <30.0	0.04 (−0.09 to 0.17)	1.04	0.14 (0.03 to 0.25)	1.15
30.0 to <35.0	0.50 (0.35 to 0.65)	1.65	0.57 (0.44 to 0.69)	1.76
35.0 to <40.0	0.87 (0.67 to 1.07)	2.39	0.99 (0.81 to 1.16)	2.68
≥40.0	1.15 (0.85 to 1.45)	3.16	1.19 (0.95 to 1.44)	3.29
Unknown	0.36 (0.13 to 0.58)	1.43	0.36 (0.17 to 0.54)	1.43
High blood pressure	0.39 (0.27 to 0.51)	1.48	0.36 (0.26 to 0.45)	1.43
Self-rated health				
Good	NA	NA	NA	NA
Poor or fair	0.12 (−0.02 to 0.26)	1.13	0.12 (−0.01 to 0.24)	1.12
Very good or excellent	−0.33 (−0.43 to −0.22)	0.72	−0.29 (−0.38 to −0.20)	0.75

Abbreviations: BMI, body mass index (calculated as weight in kilograms divided by height in meters squared); HR, hazard ratio; ln, natural log; NA, not applicable.

**Figure 2.**
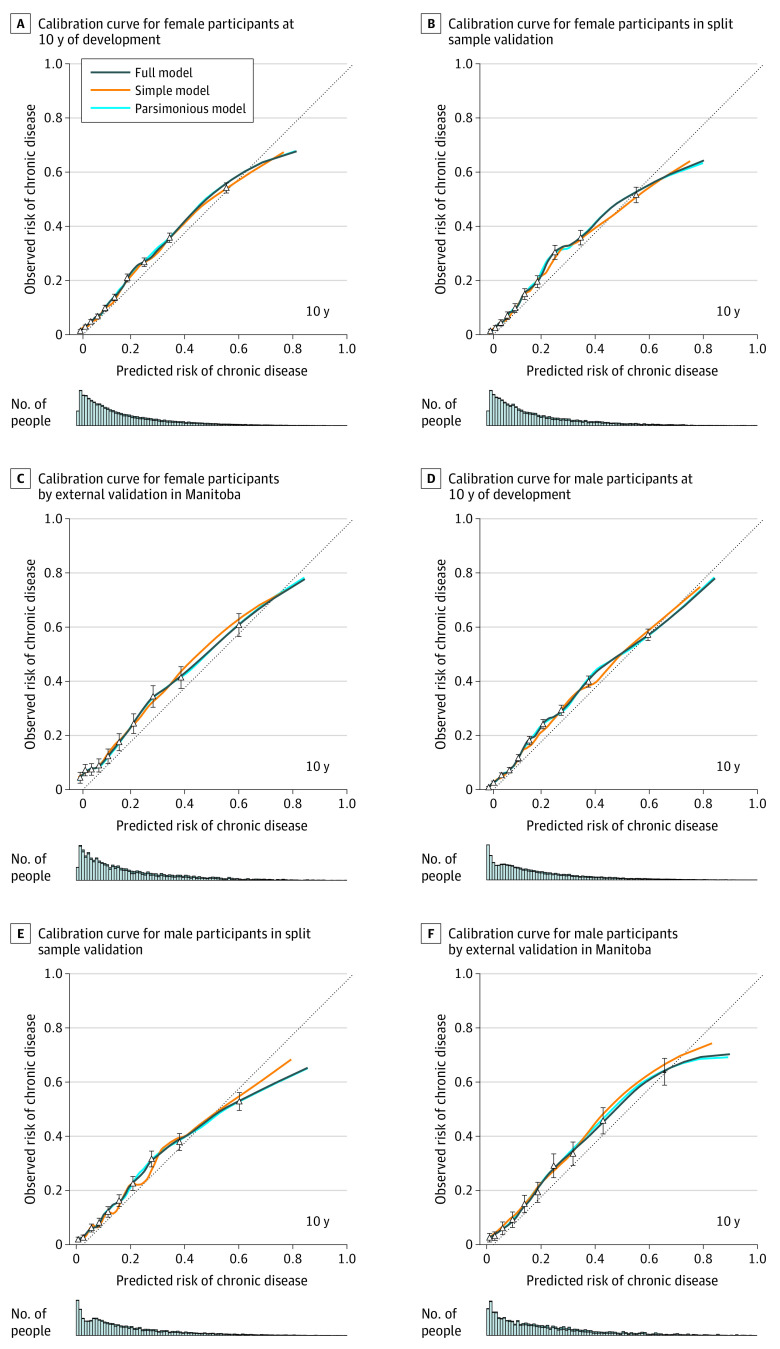
Calibration Curves for the Chronic Disease Population Risk Tool Error bars indicate 95% CIs.

The predictive performances of the development models are detailed in Table 3. In general, the full version had the best overall predictive performance (for women, Brier score, 0.087; for men, Brier score, 0.091) and discrimination (for women, discrimination slope, 0.109; for men, discrimination slope, 0.112) with the parsimonious model performing comparably (for women, Brier score: 0.087; discrimination slope, 0.109; for men, Brier score, 0.091; discrimination slope, 0.111). The simple version performed well but had worse predictive performance than the other versions (for women, Brier score: 0.088; for men, 0.092). The calibration curves (Figure 2A) indicate strong calibration up to a predicted probability of 60%, at which point the model overpredicted chronic disease risk, as indicated by the deviation of the curve from the 45° line. However, this discrepancy occurred among a very small proportion of the cohort (2.9% women and 4.1% men) and thus had minimal influence on overall calibration, as indicated by the small number of people in the bar graph below the calibration curve. By sex, the calibration curves between CDPoRT versions exhibited similar shapes.

**Table 3.** CDPoRT Predictive Performance for the Full, Simple, and Parsimonious Versions for Women and Men, by Development and Validation Settings^a^

CDPoRT version	Measures of predictive performance	Development (70%)	Bootstrap validation (development)^b^	Split sample validation (30%)	Ontario final model	Bootstrap validation (Ontario final model)^b^	Manitoba external validation	Sensitivity analysis	
								No chronic disease history	Additional chronic diseases
Full version for women, *df* = 30	Nagelkerke *R*^2^	0.160	0.159	NA	0.160	0.159	NA	NA	NA
	Brier score	0.087	NA	0.119	0.087	NA	0.128	0.090	0.322
	Harrell C index	0.779	0.778	0.777	0.767	0.779	0.752	0.774	0.596
	Discrimination slope	0.109	NA	0.096	0.089	NA	0.103	0.105	0.035
	Calibration in the large	0	0.021	−0.006	0	0.013	0.032	−0.018	0.284
	Calibration slope	1	0.994	0.994	1	0.996	0.893	0.952	0.317
Simple version for women, *df* = 14	Nagelkerke *R*^2^	0.144	0.143	NA	0.143	0.143	NA	NA	NA
	Brier score	0.088	NA	0.122	0.089	NA	0.13	0.092	0.319
	Harrell C index	0.767	0.767	0.766	0.767	0.766	0.739	0.764	0.590
	Discrimination slope	0.092	NA	0.080	0.089	NA	0.081	0.087	0.033
	Calibration in the large	0	0.010	−0.005	0	0.007	0.033	−0.016	0.280
	Calibration slope	1	0.997	1.010	1	0.998	0.894	0.972	0.293
Parsimonious version for women, *df* = 24	Nagelkerke *R*^2^	0.160	0.158	NA	0.159	0.158	NA	NA	NA
	Brier score	0.087	NA	0.119	0.088	NA	0.128	0.090	0.322
	Harrell C index	0.779	0.778	0.776	0.779	0.778	0.752	0.774	0.596
	Discrimination slope	0.109	NA	0.095	0.104	NA	0.102	0.104	0.035
	Calibration in the large	0	0.017	−0.005	0	0.012	0.03	−0.018	0.284
	Calibration slope	1	0.995	0.994	1	0.997	0.885	0.951	0.316
Full version for men *df* = 30	Nagelkerke *R*^2^	0.178	0.176	NA	0.169	0.168	NA	NA	NA
	Brier score	0.091	NA	0.091	0.091	NA	0.129	0.092	0.297
	Harrell C index	0.783	0.782	0.769	0.780	0.779	0.775	0.775	0.618
	Discrimination slope	0.112	NA	0.103	0.106	NA	0.121	0.106	0.049
	Calibration in the large	0	0.021	−0.008	0	0.015	0.013	−0.024	0.244
	Calibration slope	1	0.994	0.865	1	0.996	1.007	0.946	0.331
Simple version for men *df* = 15	Nagelkerke *R*^2^	0.159	0.176	NA	0.155	0.154	NA	NA	NA
	Brier score	0.092	NA	0.091	0.092	NA	0.129	0.093	0.294
	Harrell C index	0.773	0.773	0.765	0.771	0.771	0.766	0.768	0.614
	Discrimination slope	0.093	NA	0.095	0.092	NA.	0.099	0.093	0.045
	Calibration in the large	0	0.010	−0.003	0	0.006	0.021	−0.023	0.242
	Calibration slope	1	0.997	0.924	1	0.998	1.004	0.971	0.319
Parsimonious version for men *df* = 25	Nagelkerke *R*^2^	0.177	0.176	NA	0.168	0.167	NA	NA	NA
	Brier score	0.091	NA	0.091	0.091	NA	0.129	0.092	0.297
	Harrell C index	0.783	0.782	0.769	0.780	0.779	0.775	0.775	0.618
	Discrimination slope	0.111	NA	0.103	0.105	NA	0.120	0.105	0.049
	Calibration in the large	0	0.018	−0.003	0	0.011	0.017	−0.024	0.244
	Calibration slope	1	0.995	0.866	1	0.997	1.006	0.948	0.332

^a^ As a reference, the predictive performance measures of the female model with all predictors were Nagelkerke *R*^2^ = 0.154, Brier score = 0.088, Harrell C index = 0.775, and discrimination slope = 0.106. The predictive performance measures of the male model with all predictors were Nagelkerke *R*^2^ = 0.169, Brier score = 0.093, Harrell C index = 0.779, and discrimination slope = 0.107.

^b^ Some statistics were not calculated during bootstrap validation (rms package).

### Bootstrap Validation of the Ontario Development Cohort and Split Sample Validation

For both sexes, the baseline characteristics between the 70% sample and 30% sample were similar (standardized differences, <0.03). For model performance, the bootstrap validation and split sample validation results were similar for women and men (Table 3). For example, the C-indices for the bootstrap validation and split sample validation for the parsimonious model were 0.778 and 0.776 in women and 0.782 and 0.769 in men. The shapes of the calibration curves were also similar (Figure 2B).

### Ontario Final Model

The Ontario final models were estimated (Table 2), and each version exhibited similar overall predictive performance and discrimination compared with their development and split sample validation counterparts. For example, in the female parsimonious model, the Brier score was 0.088 (final) vs 0.087 (development) and the discrimination slope was 0.104 (final) vs 0.109 (development). The calibration curves showed a well-fit model for predicted probabilities up to 60%, with overfitting above this point (eFigure 3 in the Supplement). Bootstrap validation suggested high reproducibility.

### External Validation in Manitoba

For a given female CDPoRT version, the Brier score and calibration in the large were generally greater in the Manitoba external validation vs Ontario development and validation (eg, full version, Brier score: 0.128 vs 0.087 and 0.119; calibration in the large: 0.032 vs 0 and −0.006) (Table 3). Harrell C index and the calibration slope tended to be smaller, whereas the discrimination slope was similar in Manitoba vs Ontario (eg, parsimonious version, Harrell C index: 0.752 vs 0.779 and 0.778; calibration slope: 0.885 vs 1 vs 0.995; discrimination slope: 0.102 vs 0.109). For a given male CDPoRT version, Harrell C index, discrimination slope, calibration in the large, and the calibration slope were similar vs the Ontario settings (full version: Harrell C index: 0.775 vs 0.783 vs 0.769; discrimination slope: 0.121 vs 0.112 vs 0.103; calibration in the large: 0.013 vs 0 vs −0.008; calibration slope: 1.007 vs 1 vs 0.865). For both sexes, the Manitoba calibration plots had a shape similar to that of the Ontario calibration plots (Figure 2C).

### Sensitivity Analysis

The first sensitivity analysis examined the performance of CDPoRT among individuals who did not self-report a history of chronic disease in Ontario (n = 135 085; 75 880 [56.2%] women). The predictive performance was similar to the other results (eg, full version for women, Brier score: 0.090; full version for men, Brier score: 0.092) (Table 3; eFigure 4 in the Supplement). The second sensitivity analysis treating death as a competing risk found the subdistribution hazard ratios similar to the cause-specific hazard ratios (eTable 3 in the Supplement). The third sensitivity analysis conducted among individuals free of the original 6 chronic diseases and 9 others at baseline (n = 67 235; 34 729 [51.7%] women) found the model showed poor predictive performance (eg, full version for women, Brier score, 0.322; full version for men, Brier score, 0.316) (Table 3) and underpredicted chronic disease incidence when predicting 9 additional chronic diseases.

## Discussion

According to the predictive performance displayed during development and validation, CDPoRT exhibited good discrimination and calibration for population-based predictions of the first incidence of multiple chronic diseases, using routinely collected data with sensitivity analyses showing the tool’s robustness. Internal validation assessed CDPoRT’s reproducibility, whereas external validation assessed the tool’s transportability. According to these results, the application of CDPoRT in different settings should translate to robust performance.

The important predictors were age, smoking, and body mass index, which are widely known risk factors for single chronic diseases.^47^ As a major risk factor for lung cancer,^48^ cardiovascular disease,^49^ chronic obstructive pulmonary disease,^50^ and diabetes,^51^ cigarette smoking is a major predictor. Smoking has been found to be a major predictor in similar population risk algorithms for stroke,^12^ all-cause mortality,^30^ and cardiovascular disease.^13^ Body mass index was the most prognostic predictor for a similar diabetes tool.^9^ CDPoRT is unique vs other population risk algorithms because it accurately predicted the risk of the first of multiple chronic diseases in accordance with shared risk factors at the population level instead of for individual chronic diseases. A potential application of CDPoRT is to allow policy makers to identify and understand which population segments are at increased risk for a major chronic disease and to assess the contribution of socioeconomic, behavioral, and demographic risk factors to inform appropriate prevention strategies. Although CDPoRT does predict chronic disease burden, this burden is an underestimate because the tool does not consider all chronic diseases or subsequent chronic disease incidence (ie, multimorbidity).^5^

Three versions of CDPoRT were developed and validated, which enables transportability of the tool to other settings and under different data access conditions. Although the full version of CDPoRT had 11 predictors, the parsimonious version performed similarly with 9 predictors. This is in agreement with previous work that found diminishing improvements in predictive performance when including additional predictors.^52^ In accordance with these findings, we recommend the parsimonious CDPoRT version because it includes the predictors with the most predictive power but has fewer predictors overall to safeguard against overfitting. The parsimonious version requires fewer inputs, making it more user-friendly and less sensitive to changes in survey questions over time. The simple version is advantageous when limited data are available. The full version may be advantageous in predicting chronic disease risk for specific population subgroups. CDPoRT calculates chronic disease risk by using the coefficients listed in Table 2 and the formula in the eAppendix in the Supplement. CDPoRT was built for population-based prediction of chronic disease risk (ie, the general population). CDPoRT does not currently account for the extra variability that occurs at the individual level, so additional validation is needed to assess its performance at the individual level. The additional variability could be accounted for with new predictors (eg, biological, clinical factors) or more complex models with interaction terms.

There were case-mix differences between the Ontario and Manitoba cohorts. Thus, external validation provided insight into CDPoRT’s generalizability based on geographic transportability. Overall, CDPoRT’s predictive performance in Manitoba was similar, albeit slightly worse, in terms of discrimination and calibration vs its performance in Ontario, which suggests CDPoRT is transportable to other settings.

### Limitations

This study has limitations. Although CDPoRT was built with routinely collected survey data representative of most Canadian residents, indigenous peoples were not sampled. This is an important consideration because indigenous persons have a greater reported risk of chronic disease than nonindigenous persons in Canada.^53,54,55,56^ This may have been a contributing factor in the slight underperformance of CDPoRT in Manitoba because the indigenous population is proportionately larger there (15%) vs Ontario (2%).^57^ On-reserve survey data could be used to calibrate and update CDPoRT.

Another limitation is the measurement quality of the lifestyle risk factors. For example, fruit and vegetable consumption represented diet, whereas other food groups (eg, meats, dairy) and constituents (eg, sodium, fat) were not captured. However, greater frequencies of daily fruit and vegetable consumption are correlated with better dietary habits.^58^ Only physical activity associated with leisure was captured, whereas other forms (eg, transportation, work) were not. This may explain why physical inactivity was not predictive despite being a major risk factor for the chronic diseases under study.^59,60,61^ Capturing lifestyle behaviors across the entire population is difficult, time consuming, and costly, but this study capitalized on existing data by linking population-level surveys to administrative data.

The calibration curves consistently demonstrated overfitting for higher predicted risks. These individuals tended to be older and have suboptimal lifestyle habits (eg, daily smoking, high body mass index). They appear to represent survivors who, despite these habits, do not develop a chronic disease. CDPoRT may be missing a predictor that improves calibration in this region. For example, the Canadian Community Health Survey lacks genetic information, such as family history of chronic disease, which has been an important predictor in other models.^62^ Capturing genetic risk of chronic disease may offset the overfitting for this small subgroup because these individuals might not have a lower genetic risk for chronic disease. Despite overfitting in this subgroup, CDPoRT accurately predicts chronic disease risk for most of the population (approximately 95%), using routinely collected data that are less costly and easier to assess than clinical data.

## Conclusions

To our knowledge, CDPoRT is the first population risk algorithm that accurately predicts the first incidence of major chronic disease in adults, using routinely collected information on modifiable lifestyle risk factors during 10 years. CDPoRT has great potential applications for health policy makers in planning initiatives and supporting decision-making at the population level.

## Notes

Supplement. **eAppendix. **Calculating CDPoRT Probabilities **eFigure 1. **Log Baseline Cumulative Hazard Function of the Royston-Parmar Model Using 6 Knots and All Predictors, by Sex **eFigure 2. **Cohort Inclusion and Exclusion Criteria for the Ontario and Manitoba Cohort **eFigure 3. **Calibration Curves at 10 Years for the Sensitivity Analysis of Women and Men in Ontario Who Did Not Self-report a History of Chronic Disease, by Model Version **eFigure 4. **Calibration Curves at 10 Years for the Sensitivity Analysis of 9 Additional Chronic Disease Outcomes for Women and Men, by Model Version **eTable 1. **Definitions for Measures of Predictive Performance **eTable 2. **Starting and Final Predictor Specifications, by Sex **eTable 3. **Fine-Gray Model of Time-to-First Chronic Disease With Death as a Competing Risk Using the Parsimonious Version of CDPoRT, by Sex **eReferences.**
