# Supplementary material for: Development and Validation of the Chronic Disease Population Risk Tool (CDPoRT) to Predict Incidence of Adult Chronic Disease
Source: JAMA Netw Open. 2020 Jun 4;3(6):e204669. doi: 10.1001/jamanetworkopen.2020.4669 (PMC7273197; doi:10.1001/jamanetworkopen.2020.4669)
Supplement: Supplement. — eAppendix. Calculating CDPoRT Probabilities eFigure 1. Log Baseline Cumulative Hazard Function of the Royston-Parmar Model Using 6 Knots and All Predictors, by Sex eFigure 2. Cohort Inclusion and Exclusion Criteria for the Ontario and Manitoba Cohort eFigure 3. Calibration Curves at 10 Years for the Sensitivity Analysis of Women and Men in Ontario Who Did Not Self-report a History of Chronic Disease, by Model Version eFigure 4. Calibration Curves at 10 Years for the Sensitivity Analysis of 9 Additional Chronic Disease Outcomes for Women and Men, by Model Version eTable 1. Definitions for Measures of Predictive Performance eTable 2. Starting and Final Predictor Specifications, by Sex eTable 3. Fine-Gray Model of Time-to-First Chronic Disease With Death as a Competing Risk Using the Parsimonious Version of CDPoRT, by Sex eReferences. [file jamanetwopen-3-e204669-s001.pdf]

Supplementary Online Content

Ng R, Sutradhar R, Kornas K, et al. Development and validation of the Chronic Disease Population Risk Tool (CDPoRT) to predict incidence of adult chronic disease. *JAMA Netw Open*. 2020;3(6):e204669. doi:10.1001/jamanetworkopen.2020.4669

eAppendix. Calculating CDPoRT Probabilities

eFigure 1. Log Baseline Cumulative Hazard Function of the Royston-Parmar Model Using 6 Knots and All Predictors, by Sex

eFigure 2. Cohort Inclusion and Exclusion Criteria for the Ontario and Manitoba Cohort

eFigure 3. Calibration Curves at 10 Years for the Sensitivity Analysis of Women and Men in Ontario Who Did Not Self-report a History of Chronic Disease, by Model Version

eFigure 4. Calibration Curves at 10 Years for the Sensitivity Analysis of 9 Additional Chronic Disease Outcomes for Women and Men, by Model Version

eTable 1. Definitions for Measures of Predictive Performance

eTable 2. Starting and Final Predictor Specifications, by Sex

eTable 3. Fine-Gray Model of Time-to-First Chronic Disease With Death as a Competing Risk Using the Parsimonious Version of CDPoRT, by Sex

eReferences.

This supplementary material has been provided by the authors to give readers additional information about their work.

© 2020 Ng R et al. *JAMA Network Open.*

eAppendix. Calculating CDPoRT Probabilities

CDPoRT can calculate the probability of an individual being chronic disease-free ($Pr_{CD\text{-}free}$) or the probability of having a chronic disease (Pr_{CD}) using the Weibull PH coefficients listed in **Table 3** using the following formula:

$$\lambda = exp\ (\beta_0 + \beta_i x_i)$$

$$Pr_{CD\text{-}free} = exp\ (-\lambda t^{\gamma})$$

$$Pr_{CD} = 1 - Pr_{CD-free}$$

where λ is the linear predictor as determined from the intercept (β_0) and the self-reported characteristics of the individual (x_i) multiplied by their respective coefficients (β_i) from the Weibull PH model; *t* is the follow-up time at which the prediction is desired, in years; and γ is the shape parameter of the model. To determine the predicted number of incident chronic disease cases in the population, the predicted probabilities of chronic disease for each individual are summed together. This total can be divided by the total population size to get a predicted incidence.

Restricted cubic splines can be expressed as a difference of cubes. In this expression, a variable will have multiple terms. For example, age will have multiple values (z_j) to multiple by each of the coefficients (S_j) (**Table 2**) to determine its linear predictor (λ_{age}). The table below can be used to calculate the age linear predictor. An example is also provided

$$\lambda_{age} = exp\ (S_j z_j)$$

$$\lambda_{age\ 38,\ female} = exp\ (S_1 z_1 + S_2 z_2 + S_3 z_3)$$

$$\lambda_{age\ 38,\ female} = \exp\big(0.13(-7) + (-0.26)(1.1574) + 0.51(0.0003)\big)$$

$$\lambda_{age\ 38,\ female} = -1.21$$

Age	Female term 1 value (z_1)	Female term 2 value (z_2)	Female term 3 value (z_3)	Male term 1 value (z_1)	Male term 2 value (z_2)	Male term 3 value (z_3)	Male term 4 value (z_4)
20	-25	0	0	-25	0	0	0
21	-24	0	0	-24	0	0	0
22	-23	0	0	-23	0	0	0
23	-22	0	0	-22	0	0	0
24	-21	0.0003	0	-21	0.003	0	0
25	-20	0.0027	0	-20	0.011	0	0
26	-19	0.0093	0	-19	0.027	0	0
27	-18	0.0219	0	-18	0.052	0	0
28	-17	0.0429	0	-17	0.09	0	0
29	-16	0.0741	0	-16	0.143	0	0
30	-15	0.1176	0	-15	0.213	0	0
31	-14	0.1756	0	-14	0.304	0	0
32	-13	0.25	0	-13	0.416	0	0
33	-12	0.3429	0	-12	0.554	0	0
34	-11	0.4564	0	-11	0.72	0	0
35	-10	0.5926	0	-10	0.915	0.0004	0
36	-9	0.7534	0	-9	1.143	0.0033	0
37	-8	0.941	0	-8	1.406	0.0112	0
38	-7	1.1574	0.0003	-7	1.706	0.0267	0
39	-6	1.4047	0.0027	-6	2.046	0.0521	0

© 2020 Ng R et al. *JAMA Network Open.*

Age	Female term 1 value (z_1)	Female term 2 value (z_2)	Female term 3 value (z_3)	Male term 1 value (z_1)	Male term 2 value (z_2)	Male term 3 value (z_3)	Male term 4 value (z_4)
40	-5	1.6848	0.0093	-5	2.429	0.09	0
41	-4	2	0.0219	-4	2.857	0.1429	0
42	-3	2.3522	0.0429	-3	3.332	0.2132	0
43	-2	2.7435	0.0741	-2	3.857	0.3036	0
44	-1	3.1759	0.1176	-1	4.435	0.4165	0.0004
45	0	3.6516	0.1756	0	5.067	0.5544	0.0033
46	1	4.1725	0.25	1	5.758	0.7197	0.0112
47	2	4.7407	0.3429	2	6.508	0.915	0.0267
48	3	5.3584	0.4564	3	7.32	1.1429	0.0521
49	4	6.0274	0.5926	4	8.198	1.4057	0.09
50	5	6.75	0.7534	5	9.143	1.706	0.1429
51	6	7.5281	0.941	6	10.158	2.0462	0.2132
52	7	8.3639	1.1574	7	11.245	2.429	0.3036
53	8	9.2593	1.4047	8	12.408	2.8567	0.4165
54	9	10.2156	1.6843	9	13.648	3.3319	0.5544
55	10	11.2311	1.9954	10	14.966	3.8562	0.719
56	11	12.3032	2.3368	11	16.36	4.4276	0.9095
57	12	13.4294	2.7069	12	17.825	5.043	1.1243
58	13	14.6069	3.1045	13	19.355	5.6996	1.3618
59	14	15.8333	3.5281	14	20.947	6.3944	1.6202
60	15	17.1061	3.9765	15	22.595	7.1245	1.8981
61	16	18.4225	4.4481	16	24.294	7.8869	2.1937
62	17	19.7801	4.9417	17	26.041	8.6787	2.5055
63	18	21.1763	5.4559	18	27.83	9.497	2.8319
64	19	22.6085	5.9893	19	29.657	10.3388	3.1712
65	20	24.0741	6.5405	20	31.516	11.2012	3.5218
66	21	25.5706	7.1081	21	33.404	12.0812	3.8821
67	22	27.0953	7.6909	22	35.315	12.976	4.2505
68	23	28.6458	8.2874	23	37.246	13.8825	4.6254
69	24	30.2195	8.8962	24	39.19	14.7978	5.0051
70	25	31.8137	9.516	25	41.144	15.7189	5.388
71	26	33.4259	10.1454	26	43.102	16.6431	5.7726
72	27	35.0536	10.783	27	45.061	17.5677	6.1574
73	28	36.6941	11.4275	28	47.02	18.4923	6.5423
74	29	38.3449	12.0775	29	48.98	19.4169	6.9271
75	30	40.0034	12.7316	30	50.939	20.3415	7.312
76	31	41.6671	13.3884	31	52.898	21.2661	7.6968
77	32	43.3333	14.0466	32	54.857	22.1908	8.0816
78	33	45	14.7051	33	56.816	23.1154	8.4665
79	34	46.6667	15.3635	34	58.776	24.04	8.8513
80	35	48.3333	16.0219	35	60.735	24.9646	9.2362
81	36	50	16.6804	36	62.694	25.8892	9.621
82	37	51.6667	17.3388	37	64.653	26.8138	10.0058
83	38	53.3333	17.9973	38	66.612	27.7384	10.3907
84	39	55	18.6557	39	68.571	28.6631	10.7755
85	40	56.6667	19.3141	40	70.531	29.5877	11.1603

© 2020 Ng R et al. *JAMA Network Open.*

Age	Female term 1 value (z_1)	Female term 2 value (z_2)	Female term 3 value (z_3)	Male term 1 value (z_1)	Male term 2 value (z_2)	Male term 3 value (z_3)	Male term 4 value (z_4)
86	41	58.3333	19.9726	41	72.49	30.5123	11.5452
87	42	60	20.631	42	74.449	31.4369	11.93
88	43	61.6667	21.2894	43	76.408	32.3615	12.3149
89	44	63.3333	21.9479	44	78.367	33.2861	12.6997
90	45	65	22.6063	45	80.327	34.2107	13.0845
91	46	66.6667	23.2647	46	82.286	35.1354	13.4694
92	47	68.3333	23.9232	47	84.245	36.06	13.8542
93	48	70	24.5816	48	86.204	36.9846	14.2391
94	49	71.6667	25.2401	49	88.163	37.9092	14.6239
95	50	73.3333	25.8985	50	90.122	38.8338	15.0087
96	51	75	26.5569	51	92.082	39.7584	15.3936
97	52	76.6667	27.2154	52	94.041	40.683	15.7784
98	53	78.3333	27.8738	53	96	41.6077	16.1633
99	54	80	28.5322	54	97.959	42.5323	16.5481
100	55	81.6667	29.1907	55	99.918	43.4569	16.9329
101	56	83.3333	29.8491	56	101.878	44.3815	17.3178
102	57	85	30.5075	57	103.837	45.3061	17.7026
103	58	86.6667	31.166	58	105.796	46.2307	18.0875
104	59	88.3333	31.8244	59	107.755	47.1554	18.4723
105	60	90	32.4829	60	109.714	48.08	18.8571
106	61	91.6667	33.1413	61	111.673	49.0046	19.242
107	62	93.3333	33.7997	62	113.633	49.9292	19.6268
108	63	95	34.4582	63	115.592	50.8538	20.0117
109	64	96.6667	35.1166	64	117.551	51.7784	20.3965
110	65	98.3333	35.775	65	119.51	52.703	20.7813

© 2020 Ng R et al. *JAMA Network Open.*

eFigure 1. Log Baseline Cumulative Hazard Function of the Royston-Parmar Model Using 6 Knots and All Predictors, by Sex

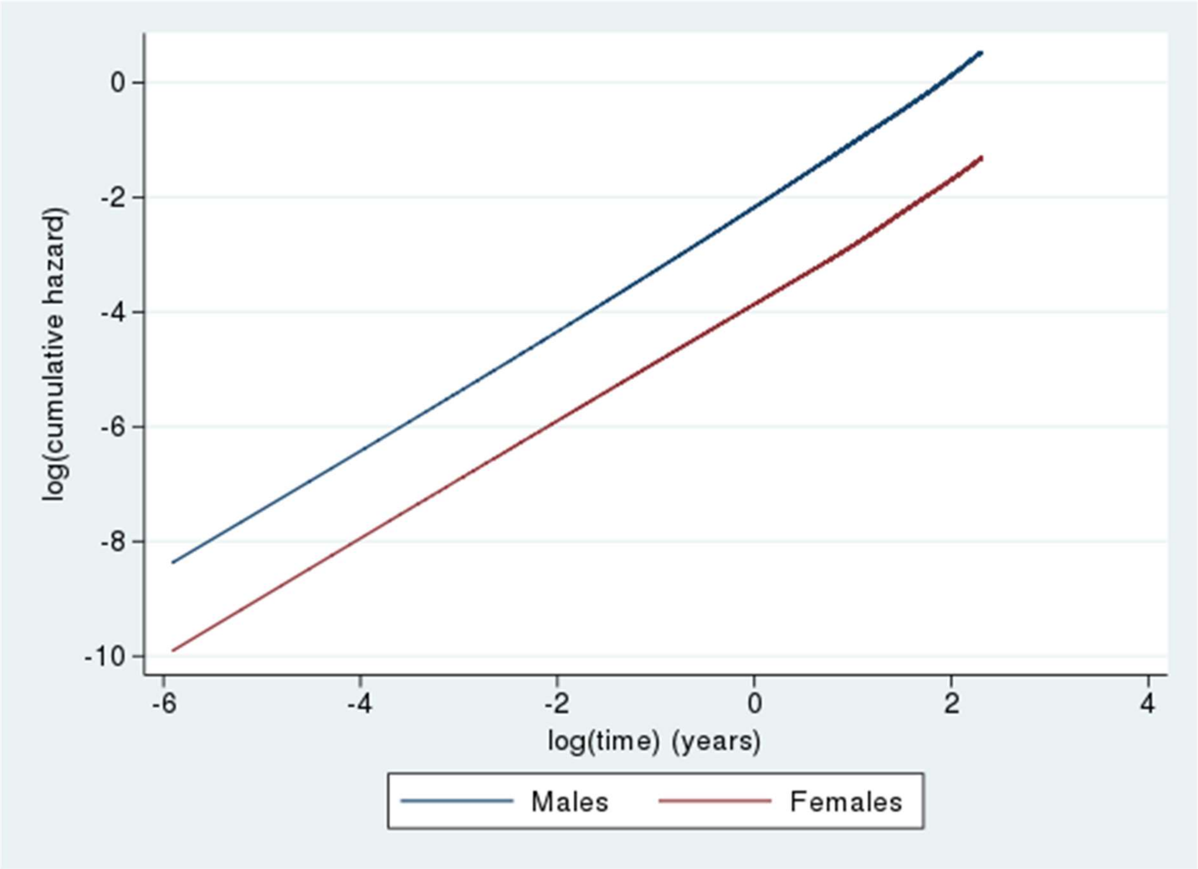

© 2020 Ng R et al. *JAMA Network Open.*

eFigure 2. Cohort Inclusion and Exclusion Criteria for the Ontario (Left) and Manitoba (Right) Cohort

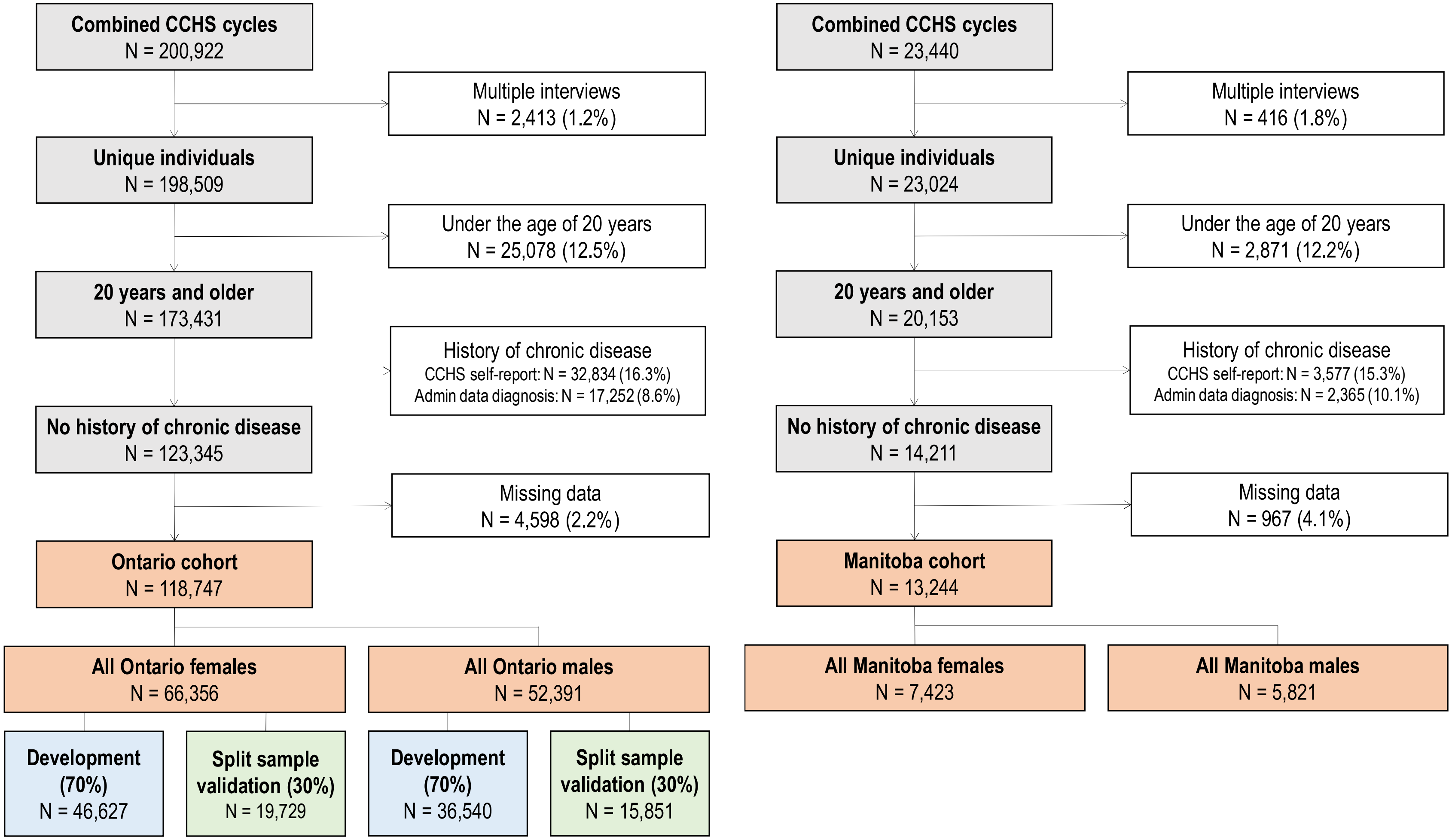

© 2020 Ng R et al. *JAMA Network Open.*

eFigure 3. Calibration Curves at 10 Years for the Sensitivity Analysis of Women (Left) and Men (Right) in Ontario Who Did Not Self-report a History of Chronic Disease, by Model Version

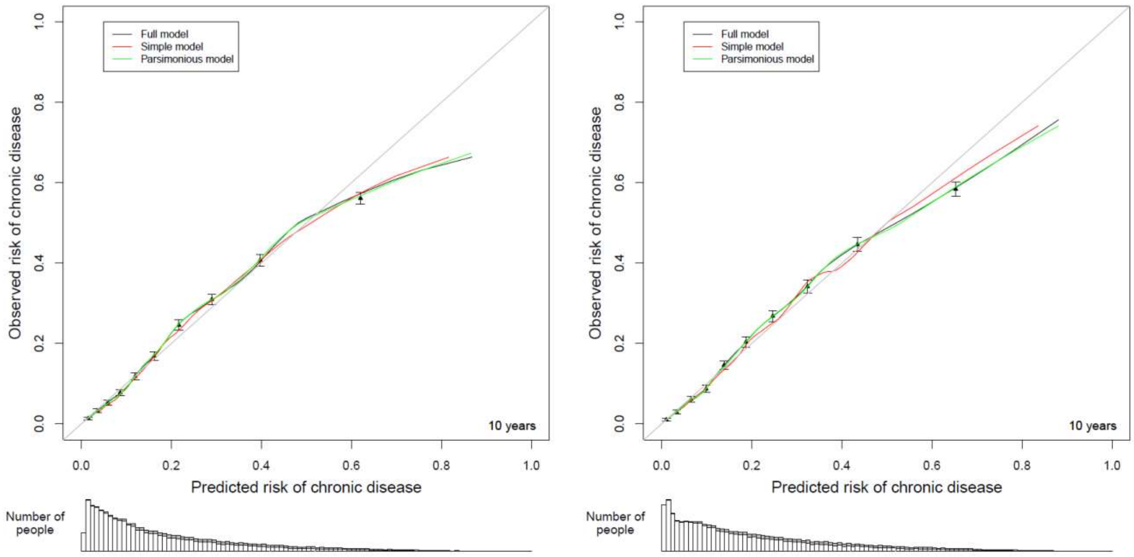

© 2020 Ng R et al. *JAMA Network Open.*

eFigure 4. Calibration Curves at 10 Years for the Sensitivity Analysis of 9 Additional Chronic Disease Outcomes for Women (Left) and Men (Right), by Model Version

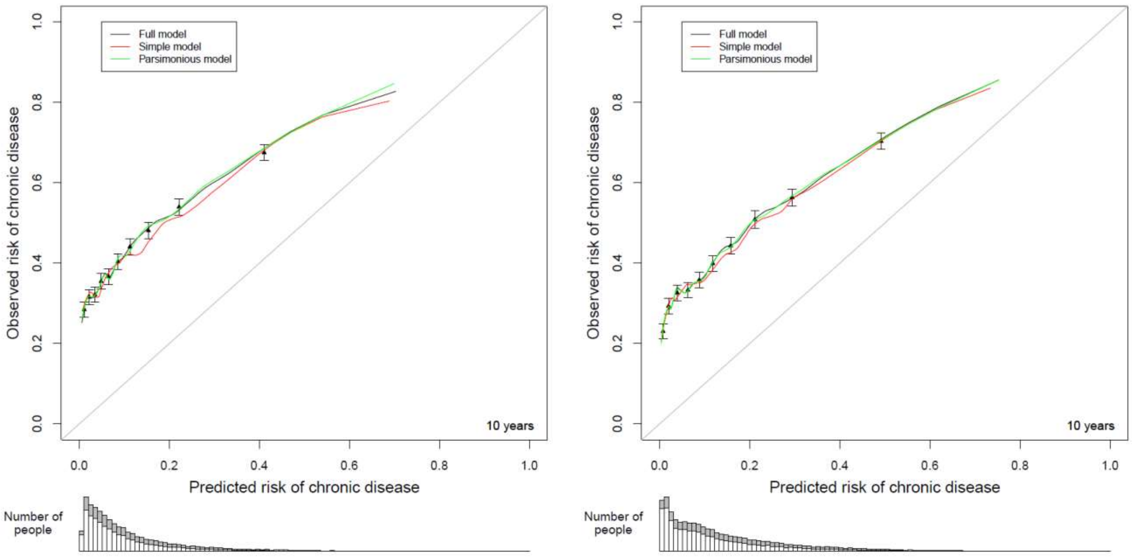

The error bars indicate 95% confidence intervals.

© 2020 Ng R et al. *JAMA Network Open.*

eTable 1. Definitions for Measures of Predictive Performance

Measures of predictive performance	Description
Overall measures	The amount of variability accounted for by the model.
Nagelkerke's R^2	Coefficient of determination that indicates the amount of variance explained by the predictors[1].
Brier score	Mean-squared prediction error[2,3].
Discrimination	The ability to distinguish between those with and without the event[4].
Harrell's concordance index (c-index)	The probability that from a randomly selected pairs of individuals, the individual with the shorter survival time has the higher predicted risk[5].
Discrimination slope	Mean difference in the predicted probabilities between those with the event and those without the event at a particular time point[4,6].
Calibration	The agreement between predicted probabilities and observed outcomes[7]. Calibration is measured at a specific time point.
Calibration curve	The calibration curve indicates the overall calibration of the model across all predicted probabilities visually[8]. The calibration curve also has a bar graph below that indicates the frequency of observations by predicted risk.
Calibration intercept	The difference in the mean observed risk and the mean predicted risk)[9].
Calibration slope	A measure of the overall calibration of the model across all predicted probabilities[9].

Time-specific measure are measures that capture all cases up to time *t* (i.e. cumulative cases) and non-cases that have not yet had the outcome but are still at-risk (i.e. dynamic controls)[10].

© 2020 Ng R et al. *JAMA Network Open.*

eTable 2. Starting and Final Predictor Specifications, by Sex

Predictor	Starting specification	Final specification (female full version)	Final specification (male full version)
**Modifiable lifestyle risk factors**			
Alcohol consumption	Non-drinker<br>Light drinker<br>Moderate drinker<br>Heavy drinker	Unchanged	Unchanged
Cigarette smoking	Never smoked<br>Light smoker<br>Heavy smoker<br>Former light smoker<br>Former heavy smoker<br>Former smoker, unknown amount	Never smoked<br>Always an occasional smoker<br>Current occasional smoker, former daily smoker<br>Daily smoker<br>Former occasional smoker<br>Former daily smoker	Never smoked<br>Always an occasional smoker<br>Current occasional smoker, former daily smoker<br>Daily smoker<br>Former occasional smoker<br>Former daily smoker
Daily fruit and vegetable consumption	0 to 3 times<br>3 to 6 times<br>6 or more times	Unchanged	Unchanged
Physical activity quartile	Quartile 1 (lowest)<br>2<br>3<br>Quartile 4 (highest)	Excluded	Excluded
**Sociodemographic factors**			
Age	20 to 34 years<br>35 to 44 years<br>45 to 54 years<br>55 to 64 years<br>65 to 74 years<br>75 to 84 years<br>85+ years	Restricted cubic spline centered at age 45 years with 4 knots (at 23, 37, 53 and 77 years)	Restricted cubic spline centered at age 45 years with 5 knots (at 22, 34, 43, 54 and 71 years)
Ethnicity	White / visible minority	Unchanged	Unchanged
Immigration status	Canadian-born<br>Non-recent immigrant (10+ years)<br>Recent immigrant (<10 years)	Excluded	Excluded
Income quintile	Quintile 1 (lowest)<br>2<br>3<br>4<br>Quintile 5 (highest)<br>Unknown income	Excluded	Low income (Q 1)<br>Not low income (Q2 to Q5)<br>Unknown income
Education	Less than secondary school graduation<br>Secondary school graduation<br>Post-secondary education (including partial)	No post-secondary education<br>Post-secondary education (including partial)	Excluded
Marital status	Single, never married<br>Domestic partner (married or common-law)<br>Widowed, separated or divorced	Unchanged	Excluded
**Other health-related factors**			

© 2020 Ng R et al. *JAMA Network Open.*

Predictor	Starting specification	Final specification (female full version)	Final specification (male full version)
Asthma	Yes / no	Unchanged	Unchanged
Body mass index (BMI), kg/m2	Underweight (<18.5)<br>Normal weight (18.5 to 25)<br>Overweight (25 to 30)<br>Obese class 1 (30 to 35)<br>Obese class 2 (35 to 40)<br>Obese class 3 (>40)<br>Unknown BMI	Unchanged	Unchanged
High blood pressure	Yes / no	Unchanged	Unchanged
Household secondhand smoke	Yes / no	Excluded	Excluded
Self-rated health	Poor or fair<br>Good<br>Very good or excellent	Unchanged	Unchanged
Self-rated stress	Not at all stressful<br>Not very stressful<br>A bit stressful<br>Quite a bit or extremely stressful	Unchanged	Unchanged

All responses are based on individual self-report.

© 2020 Ng R et al. *JAMA Network Open.*

eTable 3. Fine-Gray Model of Time-to-First Chronic Disease With Death as a Competing Risk Using the Parsimonious Version of CDPoRT, by Sex

Variable			Female			Male		
			sdHR (95% CI)		P-value	sdHR (95% CI)		P-value
Alcohol consumption	Heavy drinker	Light drinker	1.29	(1.00, 1.67)	0.049	0.89	(0.77, 1.03)	0.111
	Moderate drinker		1.10	(0.97, 1.26)	0.131	0.99	(0.88, 1.12)	0.870
	Non-drinker		1.38	(1.25, 1.53)	<0.001	1.21	(1.07, 1.35)	0.002
Cigarette smoking	Heavy drinker	Never smoked	1.31	(0.90, 1.89)	0.158	1.06	(0.73, 1.53)	0.762
	Moderate drinker		1.84	(1.46, 2.32)	<0.001	1.42	(1.13, 1.78)	0.002
	Non-drinker		2.90	(2.62, 3.21)	<0.001	2.30	(2.05, 2.58)	<0.001
	Former occasional smoker		0.85	(0.76, 0.96)	0.007	1.01	(0.86, 1.19)	0.888
	Former daily smoker		1.21	(1.09, 1.35)	<0.001	1.18	(1.05, 1.32)	0.005
Daily fruit and vegetable consumption	3 to 6 times	0 to 3 times	0.93	(0.85, 1.02)	0.120	0.93	(0.85, 1.02)	0.130
	6+ times		0.88	(0.78, 0.98)	0.021	0.85	(0.75, 0.97)	0.016
Age	Age (spline term 1)		1.13	(1.11, 1.16)	<0.001	1.21	(1.16, 1.26)	<0.001
	Age (spline term 2)		0.79	(0.74, 0.85)	<0.001	0.66	(0.53, 0.82)	<0.001
	Age (spline term 3)		1.61	(1.38, 1.88)	<0.001	2.57	(1.28, 5.15)	0.008
	Age (spline term 4)		--	--	--	0.59	(0.30, 1.18)	0.134
Visible minority			1.41	(1.25, 1.61)	<0.001	1.35	(1.19, 1.52)	<0.001
Asthma			1.46	(1.30, 1.65)	<0.001	1.32	(1.12, 1.56)	0.001
BMI	Underweight	Normal weight	0.82	(0.55, 1.24)	0.350	1.53	(0.97, 2.43)	0.070
	Overweight		1.46	(1.32, 1.62)	<0.001	1.16	(1.04, 1.30)	0.007
	Obese class 1		1.87	(1.67, 2.10)	<0.001	1.78	(1.56, 2.02)	<0.001
	Obese class 2		2.85	(2.38, 3.41)	<0.001	2.70	(2.26, 3.22)	<0.001
	Obese class 3		3.24	(2.66, 3.93)	<0.001	3.33	(2.63, 4.22)	<0.001
	Unknown BMI		1.55	(1.36, 1.77)	<0.001	1.42	(1.18, 1.70)	<0.001
High blood pressure			1.40	(1.27, 1.54)	<0.001	1.44	(1.30, 1.58)	<0.001
Self-rated health	Poor/fair	Good	1.23	(1.09, 1.38)	0.001	1.10	(0.97, 1.25)	0.130
	Very good / excellent		0.83	(0.76, 0.90)	<0.001	0.75	(0.68, 0.82)	<0.001

© 2020 Ng R et al. *JAMA Network Open.*

eReferences

1. Nagelkerke NJD. A note on a general definition of the coefficient of determination. *Biometrika*. 1991;78(3):691-692. doi:10.1093/biomet/78.3.691

2. Austin PC, Pencinca MJ, Steyerberg EW. Predictive accuracy of novel risk factors and markers: A simulation study of the sensitivity of different performance measures for the Cox proportional hazards regression model. *Stat Methods Med Res*. 2017;26(3):1053-1077. doi:10.1177/0962280214567141

3. Brier GW. Verification of forecasts expersses in terms of probaility. *Mon Weather Rev*. 1950;78(1):1-3. doi:10.1126/science.27.693.594

4. Steyerberg EW, Vickers AJ, Cook NR, et al. Assessing the performance of prediction models: a framework for traditional and novel measures. *Epidemiology*. 2010;21(1):128-138. doi:10.1097/EDE.0b013e3181c30fb2

5. Rahman MS, Ambler G, Choodari-Oskooei B, Omar RZ. Review and evaluation of performance measures for survival prediction models in external validation settings. *BMC Med Res Methodol*. 2017;17(1):1-15. doi:10.1186/s12874-017-0336-2

6. Yates JF. External correspondence: Decompositions of the mean probability score. *Organ Behav Hum Perform*. 1982;30(1):132-156. doi:10.1016/0030-5073(82)90237-9

7. Moons KGM, Altman DG, Reitsma JB, et al. Transparent Reporting of a multivariable prediction model for Individual Prognosis Or Diagnosis (TRIPOD): Explanation and Elaboration. *Ann Intern Med*. 2015;162(1):W1-W73. doi:10.7326/M14-0698

8. Van Calster B, Nieboer D, Vergouwe Y, De Cock B, Pencina MJ, Steyerberg EW. A calibration hierarchy for risk models was defined: From utopia to empirical data. *J Clin Epidemiol*. 2016;74:167-176. doi:10.1016/j.jclinepi.2015.12.005

9. Steyerberg EW, Vergouwe Y. Towards better clinical prediction models: Seven steps for development and an ABCD for validation. *Eur Heart J*. 2014;35(29):1925-1931. doi:10.1093/eurheartj/ehu207

10. Liang CJ, Heagerty PJ. A risk-based measure of time-varying prognostic discrimination for survival models. *Biometrics*. 2017;73(3):725-734. doi:10.1111/biom.12628

© 2020 Ng R et al. *JAMA Network Open.*
